# Contour‐based lung dose prediction for breast proton therapy

**DOI:** 10.1002/acm2.12436

**Published:** 2018-08-23

**Authors:** Chuan Zeng, Kevin Sine, Dennis Mah

**Affiliations:** ^1^ ProCure Proton Therapy Center Somerset NJ USA

**Keywords:** breast cancer, dose prediction, radiation therapy

## Abstract

**Purpose:**

This study evaluates the feasibility of lung dose prediction based on target contour and patient anatomy for breast patients treated with proton therapy.

**Methods:**

Fifty‐two randomly selected patients were included in the cohort, who were treated to 50.4–66.4 Gy(RBE) to the left (36), right (15), or bilateral (1) breast with uniform scanning (32) or pencil beam scanning (20). Anterior‐oblique beams were used for each patient. The prescription doses were all scaled to 50.4 Gy(RBE) for the current analysis. Isotropic expansions of the planning target volume of various margins *m* were retrospectively generated and compared with isodose volumes in the ipsilateral lung. The fractional volume *V* of each expansion contour within the ipsilateral lung was compared with dose–volume data of clinical plans to establish the relationship between the margin *m* and dose *D* for the ipsilateral lung such that *V*_*D*_ = *V*(*m*). This relationship enables prediction of dose–volume *V*_*D*_ from *V*(*m*), which could be derived from contours before any plan is generated, providing a goal of plan quality. Lung *V*_20 Gy(_ _RBE__)_ and *V*_5 Gy(_ _RBE__)_ were considered for this pilot study, while the results could be generalized to other dose levels and/or other organs.

**Results:**

The actual *V*_20 Gy(_ _RBE__)_ ranged from 6% to 23%. No statistically significant difference in *V*_20 Gy(_ _RBE__)_ was found between breast irradiation and chest wall irradiation (*P* = 0.8) or between left‐side and right‐side treatment (*P* = 0.9). It was found that *V*(1.1 cm) predicted *V*_20 Gy(_ _RBE__)_ to within 5% root‐mean‐square deviation (RMSD) and *V*(2.2 cm) predicted *V*_5 Gy(_ _RBE__)_ to within 6% RMSD.

**Conclusion:**

A contour‐based model was established to predict dose to ipsilateral lung in breast treatment. Clinically relevant accuracy was demonstrated. This model facilitates dose prediction before treatment planning. It could serve as a guide toward realistic clinical goals in the planning stage.

**Abbreviations**
CT: computed tomography
GE: General Electric
IL: ipsilateral lung
MC: Monte Carlo
ME: mean error
OAR: organ at risk
PB: pencil beam
PBS: pencil beam scanning
PT: proton therapy
PTV: planning target volume
RMSD: root‐mean‐square deviation
US: uniform scanning

## 1 INTRODUCTION

Radiation dose distribution depends on target size and target location relative to organs at risk. Therefore, it is often difficult to know *a priori* what the dose to normal tissues for a patient may be achievable. This can make selecting the optimal treatment approach for a specific patient challenging. Being able to predict the dose to normal tissues prior to treatment planning can guide radiation oncologists and physicists toward realistic clinical goals in the planning stage and ensure the quality of treatment plans. This could also enable physicians at hospitals without proton capabilities to make a better‐informed referral decision or aid patient selection.

Modern radiation treatments deliver highly conformal dose to the target. As a result, the falloff isodose lines typically resemble uniform expansions of the planning target volume. We propose to predict isodose lines based on target contour and thus predict dose–volume *V*_*D*_, thereby allowing *V*_*D*_ and metrics of plan quality to be derived from contours before any plan is generated.

The treatment of breast cancer is a relatively new application of proton therapy (PT).^1^ While recent comparative treatment planning studies of PT for breast cancer have highlighted the significant advantage in heart and lung sparing as well as in target coverage over traditional photon‐based radiation techniques,^2^, ^3^ for each individual case, it is not straightforward to determine the dose–volume metrics achievable with PT without generating a treatment plan. In this pilot study, we attempt to predict ipsilateral lung (IL) *V*_20 Gy(RBE)_ and *V*_5 Gy(RBE)_ based on the contours of each individual patient.

## 2 METHODS

### 2.A Patient selection

Fifty‐two randomly selected patients recently (2016–2017) treated for breast cancer in our clinic with uniform scanning (US) or pencil beam scanning (PBS) proton therapy were included in this study, which has been reviewed by our institutional review board. The diseases were left‐sided for 36 patients, right‐sided for 15, and bilateral for 1. Eighty percent of the patients had nodal involvement; and partial breast irradiation was not considered in this study. The prescription doses varied from 50.4 Gy(RBE) to 66.4 Gy(RBE).

### 2.B Setup and delivery

Computed tomography (CT) scanning was performed for each patient at the simulation with the arms abducted above the head using a custom mold (Alpha Cradle, Smithers Medical Products, Inc., North Canton, OH, USA). Patients were immobilized in the supine position. There was no actual immobilization of the breast. The CTs were performed on a GE LightSpeed/Optima CT scanner.

The target delineation was described in our previous publication.^4^ Although controversial in proton therapy,^5^ the planning target volume (PTV) is still widely used by clinicians in evaluation of proton treatment plans (e.g., Ref. 4, 6, 7). There is also debate over the appropriate use of PTV margins for setup uncertainty and motion for breast cancer. We used a 7 mm margin medially and laterally but not posteriorly. In the distal/posterior direction, the plans were generated to cover the clinical target volumes and evaluated for range uncertainty.^8^ In addition, we did not add the margin medially in the supraclavicular region to avoid extending the PTV into the esophagus. For complete review of the uncertainty margins and their application in US and PBS, refer to our recent book chapter.^9^

Proton therapy was typically delivered with two anterior oblique *en face* fields. For US, due to the limitation in field size, matching fields were typically needed to cover the chest wall and supraclavicular nodes, with the match lines feathered.^4^

### 2.C Dose prediction

We rescaled all the initial prescription doses to 50.4 Gy(RBE) for the current analysis. Boost dose was not considered. Isotropic expansions of the PTV of various margins *m* were retrospectively generated (denoted as PTV + *m*) and compared with isodose volumes in the IL. The fractional volume *V* of each expansion contour within the IL was compared with dose–volume data of clinical plans to establish the relationship between the margin *m* and dose *D* for the IL such that *V*_*D*_ = *V*(*m*). This relationship enables prediction of dose–volume *V*_*D*_ from *V*(*m*), which could be derived from contours before any plan is generated, providing a goal of plan quality. Lung *V*_20 Gy(RBE)_ and *V*_5 Gy(RBE)_ were considered for this pilot study, while the results could be generalized to other dose levels and/or other organs. Note that *m* could be generalized to negative values, corresponding to contours isotropically shrunk from PTV.

Ten patients were randomly selected, for whom *m* was adjusted to minimize the root‐mean‐square error (RMSE) of prediction *V*(*m*) for *V*_*D*_:

(1) $$m\left(D\right):={\arg\;\min}_{m^{\prime }}\langle {\left[V\left(m^{\prime }\right)-V_{D}\right]}^{2}\rangle ,$$

where the chevron 〈 〉 denotes an average over patients. For each particular dose level *D*, patients were subsequently added to the cohort until the RMSE did not change significantly as the sample size further increased. That ensured the estimated prediction uncertainty be invariant under change of sample size; it also justified the sufficiency of the sample size of our study. The mean error,

(2) $$\mathrm{ME}=\langle V\left(m\right)-V_{D}\rangle ,$$

was also tracked as a measure of systematic error of our prediction.

The patient treated for bilateral disease was considered as two cases, one left, and one right.

## 3 RESULTS

**Figure 1:**
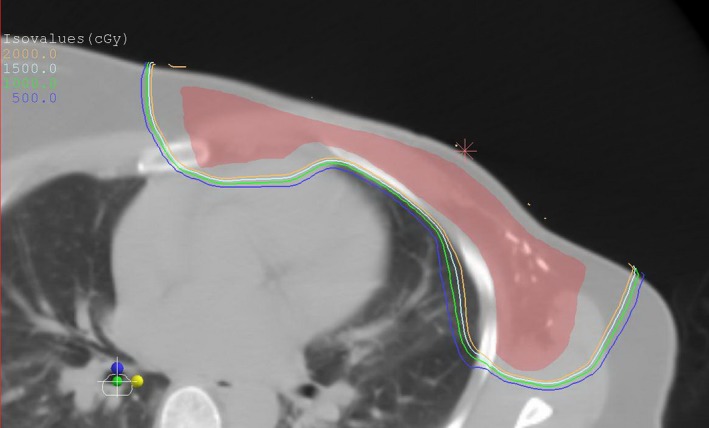
Treatment plan for a typical patient. The filled contour shown is the planning target volume.

**Figure 2:**
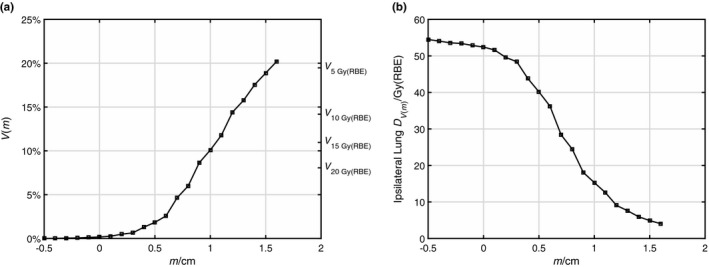
Mapping of dose–volume *V*_*D*_ (a) and volume–dose *D*_*V*_ (b) data to variable margin *m* for the patient shown in Fig. 1.

Figure 1 demonstrated the treatment plan for one typical patient. It is evident that the falloff isodose lines resemble uniform expansions of the PTV, signifying the high conformality achieved. In Fig. 2(a), the volume of intersection of PTV + *m* and IL, *V*(*m*), for that patient was plotted as a function of *m* and compared with the dose–volume *V*_*D*_ of IL in the actual treatment plan. For this particular patient, *V*(9 mm) approximated IL *V*_20 Gy(RBE)_; and *V*(16 mm) approximated IL *V*_5 Gy(RBE)_. Alternatively, volume–dose *D*_*V*_ corresponding to *V*(*m*) could be plotted against *m* [Fig. 2(b)], showing the isodose level *D* estimated by contour expansion PTV + *m*. Figure 2(b) also reflected the average dose falloff in the IL, showing an 80%–20% falloff of about 7 mm.

**Figure 3:**
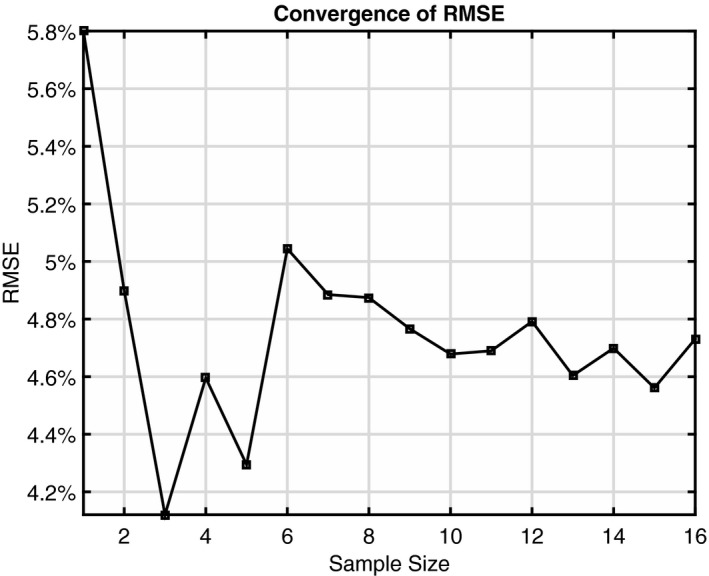
Convergence of root‐mean‐square error (RMSE) of *V*_20 Gy(_ _RBE__)_ prediction for ipsilateral lung.

**Figure 4:**
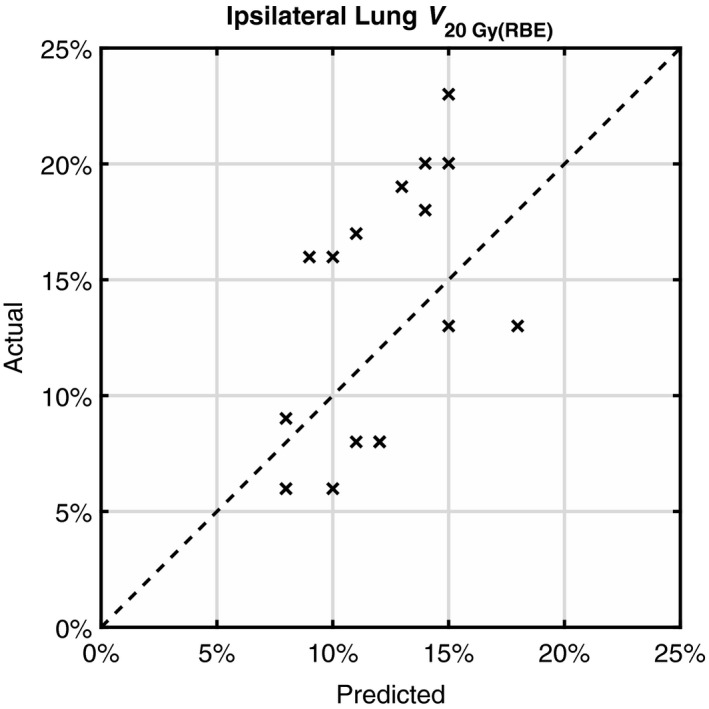
Predicted ipsilateral lung *V*_20 Gy(_ _RBE__)_ vs actual values for 16 patients. The dashed line corresponds to errorless prediction.

By minimizing RMSE for the first ten patients, it was determined that *V*(11 mm) best estimated ipsilateral *V*_20 Gy(RBE)_. The sample size increased to 16 to achieve convergence of RMSE, as shown in Fig. 3. The RMSE for *V*_20 Gy(RBE)_ prediction converged to slightly less than 5%. The SE was −2%, indicating an insignificant (compared to the RMSE of 5%) systematic underestimation. The predicted and actual *V*_20 Gy(RBE)_ values for those 16 patients were plotted in Fig. 4. The actual *V*_20 Gy(RBE)_ ranged from 6% to 23%. No statistically significant difference in *V*_20 Gy(RBE)_ was found between breast irradiation and chest wall irradiation (*P* = 0.8; two‐sample *t*‐test) or between left‐side and right‐side treatment (*P* = 0.9; two‐sample *t*‐test).

**Figure 5:**
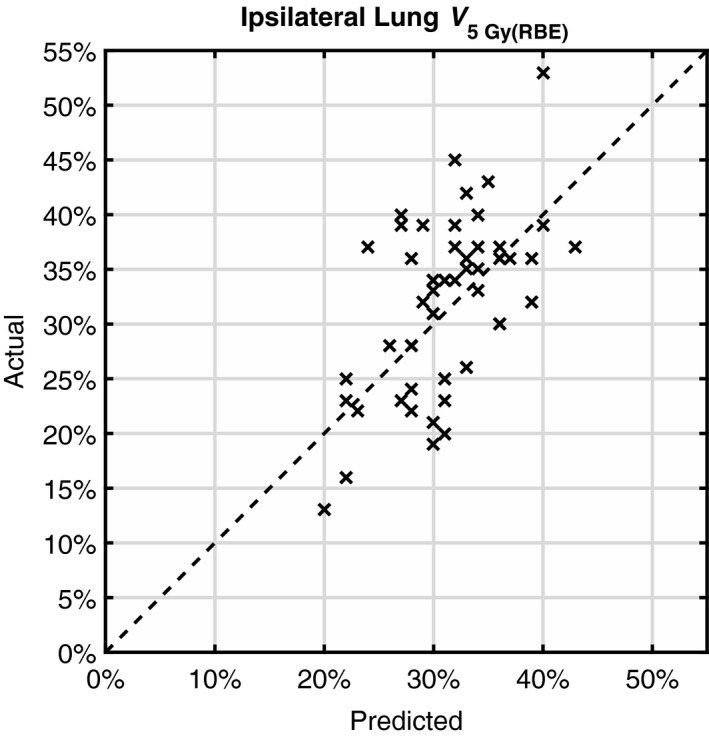
Predicted ipsilateral lung *V*_5 Gy(_ _RBE__)_ vs actual values for 52 patients. The dashed line corresponds to errorless prediction.

For the estimation of *V*_5 Gy(RBE)_, *m* was determined to be 22 mm. Convergence of RMSE to 6% was observed for the 52 patients included. The SE was −1%, indicating again an insignificant (compared to the RMSE of 6%) systematic underestimation. The predicted and actual *V*_5 Gy(RBE)_ values for those 52 patients were plotted in Fig. 5. The actual *V*_5 Gy(RBE)_ ranged from 13% to 53%.

## 4 DISCUSSION

A contour‐based model was established to predict dose to IL in breast treatment. The prediction accuracy has been demonstrated. This model facilitates dose prediction before treatment planning. It could serve as a guide toward realistic clinical goals in the planning stage.

Since the conclusion of this clinical investigation, we have been running the lung dose estimation for all our breast cancer patients before treatment planning. The model serves as a strong reference to our treatment planning goals and played an important role in ensuring plan quality. Our final dose values were all reasonably close to the model prediction.

The general methodology to develop such a model involves (a) visually estimating the distance *m* from target contour to a particular isodose line for a series of previously planned cases; (b) expanding the target by amount *m* and using it as the predicted isodose line for a number of cases to establish the model uncertainty for particular dose–volume metrics; and (c) possibly adjusting the value *m* based on a running population study until the uncertainty converges. Such models will also be useful when an estimation of the feasible benefits of proton therapy is desired, but the experience and/or resources required for treatment planning are unavailable.

Several methods previously developed to predict organs‐at‐risk (OAR) dose levels achievable with advanced photon therapies were geometric knowledge‐based.^10^, ^11^, ^12^, ^13^, ^14^ They were also developed within the contexts of (a) treatment plan quality in an attempt to detect suboptimal plans and automate the decision for replanning, and (b) providing planning objectives to initiate the treatment planning process. Most of these methods start from a quantity related to distance‐to‐target or overlap volume.

One of the strengths of our model is its simplicity. It can be applied to a new clinical case once the target contour is available and the IL is delineated. The only functions necessary are isotropic expansion, intersection, and volume calculation or voxel count. Those functions are virtually available in any contouring module. An estimation may be obtained within minutes or even seconds. In contrast, developing a clinical treatment plan can easily take hours even after the contours are ready. If programming or scripting interface is available, the model could be easily implemented as a one‐button solution.

Despite its simplicity, the model is effectively accounting for a number of patient‐specific features in estimating dose–volume metrics. Since the prediction is derived directly from patient contours, the size of target and OARs (e.g., lung), the separation between target and OARs (e.g., thickness of rib cage), shapes, and/or curvatures are all implicitly considered in the model.

In our determination of the model parameter *m*, we increased the sample size until the convergence of prediction error was achieved (Fig. 3). That means further increasing the sample size in establishing the model would not improve the prediction accuracy. In other words, the currently observed prediction error is the intrinsic limit of the model itself.

One of the limitations of the model is the lack of consideration of CT density information. Breast treatment inherently involves the interface between soft tissue, bone, and lung. If we replace the isotropic *geometric margin* expansion with *water‐equivalent margin* expansion and determine the model parameter accordingly, significant improvement in prediction accuracy may be expected. (To the best of our knowledge, this desired water‐equivalent margin expansion is not yet available in any commercial treatment planning system so far.) Further improvement may involve various machine learning approaches (e.g., Ref.^15^). However, as the complexity of modeling increases, the technical challenge of implementation may arise depending on the resources and capabilities of each clinic.

**Figure 6:**
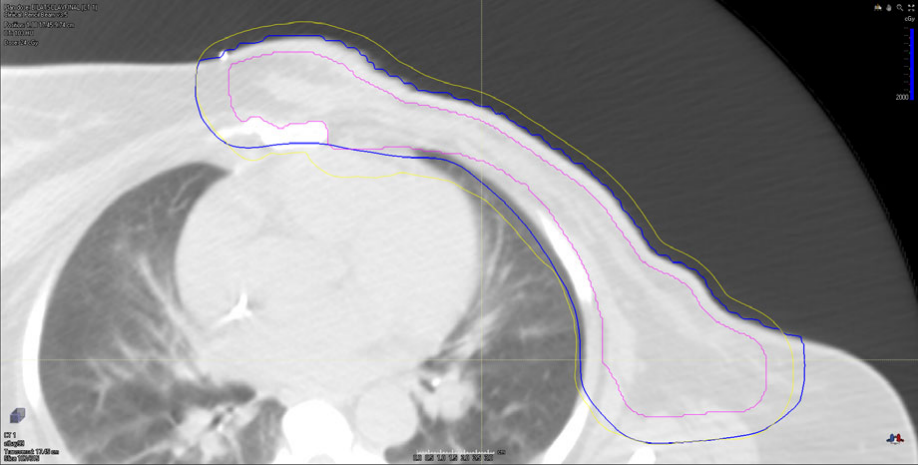
An example case showing compromised dose conformality due to desired heart sparing. The magenta contour shows the planning target volume (PTV). The yellow contour shows the predicted 20 Gy(RBE) isodose line, which is an expansion of 11 mm from the PTV. The blue contour is the actual 20 Gy(RBE) isodose line.

Another limitation of our model lies in the assumption of dose conformality. In a clinical treatment plan, indeed, dose gradient may vary in different parts of the PTV surface, depending on the combination of beams. Thus, the degree of conformality may not be uniform across the PTV surface. We effectively averaged the dose gradient around the entire PTV surface and summarized it into a single parameter *m*, disregarding the positional dependence of local dose gradient. However, there are certain clinical scenarios where dose conformality is compromised giving way to normal tissue sparing (e.g., heart, chest wall, rib). Those cases will inevitably become outliers for our model, one example of which is shown in Fig. 6. In that case, part of the PTV is underdosed in order to spare the heart, which may be a common compromise one has to make in breast cases where internal mammary nodes are involved. As shown in the figure, the medial portion of PTV is touching the 20 Gy(RBE) isodose line, indicating a cold spot as low as 20 Gy(RBE), compared to the prescription dose of 50.4 Gy(RBE). Since that compromise was not accounted for in our model, the model overestimated the lung dose. *V*_20 Gy(RBE)_ was predicted to be 18%, while the actual value was 13%. *V*_5 Gy(RBE)_ was also overestimated to be 39%, while the actual value was 32%.

The dose predictions are limited by the accuracy of dose calculation algorithm, especially in lung. Pencil beam (PB) algorithm was used in this study.^16^ The deficiencies of PB algorithm have been reported in the literature for many years.^17^, ^18^, ^19^, ^20^, ^21^ As commercial Monte Carlo (MC) dose calculation algorithms recently became available,^22^, ^23^ the need for MC algorithm was demonstrated unequivocally for lung targets.^24^ In a dosimetric comparison for ten lung cancer patients, it was shown that total lung *V*_20 Gy(RBE)_ maintained a 2% deviation across PB and MC, and similar trends were observed for *V*_10 Gy(RBE)_ and *V*_5 Gy(RBE)._ 25 For breast cancer, the clinical application of MC is still limited. The comparison of lung dose in breast treatment as computed with PB and MC warrants future investigation. As we started using MC for select cases of breast cancer, the differences in lung dose compared to US were likely within 5% and were certainly negligible compared to the interpatient anatomical variations.

The air gap may differ between US and PBS resulting changes in penumbra. On the one hand, the US range compensators are usually smaller due to a larger number of beams involved to cover the target. However, our US delivery is limited by the fixed gantry angles (inclined beam geometry; beam at 90° and 30° only; 26), while our PBS treatments for breast cases are delivered on a full gantry. Therefore, on the other hand, PBS is more flexible in choosing the optimal gantry angle and minimizing the air gap. Furthermore, the curved shape of breast targets always results in variation in air gap within the same target. No clear systematic difference was identified in air gap between US and PBS. For our study, the comparison of IL *V*_20 Gy(RBE)_ between US and PBS cases did not show statistically significant difference (*P* = 0.9; two‐sample *t*‐test). Another difference between US and PBS is the presence and absence of lateral collimation. No aperture was used for the PBS treatments in the current study, which potentially may result in larger lateral penumbra. However, the major contribution of lung dose is from the distal penumbra, which may explain the absence of significant difference in IL *V*_20 Gy(RBE)_ between US and PBS cases.

The intention of this study was to provide a patient‐specific guideline dose for lung sparing, regardless of delivery technique. Based on our analysis, there is no statistically significant difference in IL *V*_20 Gy(RBE)_ between US and PBS cases. In terms of the treatment planning process, high conformality is desired for both US and PBS plans. For our cohort, separate evaluation of the predicted IL *V*_20 Gy(RBE)_ for US and PBS, using the same parameter *m*, both yielded an RMSE of 5%. Thus, no systematic difference was observed in the prediction accuracy between US and PBS.

Of note, the high conformality achieved in our cases for the low‐dose levels is indicative of one of the advantages of proton therapy for breast cancer patients: 5 Gy(RBE) is only ~10% of prescription dose; yet the isodose line still conforms well to the PTV. It may not be the case for a different technique (e.g., parallel‐opposed beams), in which only high‐dose levels are conformed to the target.

Among the patients included in this study, 69% received boost radiation. While the boost dose was not considered in our model, it is our observation that boost radiation only increased the IL *V*_20 Gy(RBE)_ by 1%–2%, which is less than the RMSE of our prediction. For about one‐third of the cohort, the initial prescription dose was 45 Gy(RBE) or 46 Gy(RBE).

The actual IL dose should be slightly smaller than our prediction because our model was based on a prescription of 50.4 Gy(RBE). However, the difference in lung dose is not that significant. For example, for a prescription of 45 Gy(RBE), the expansion PTV + 11 mm would predict, instead of 20 Gy(RBE) isodose line, $20\;\mathrm{Gy}\left(\mathrm{RBE}\right)\times \frac{45\;\mathrm{Gy}\left(\mathrm{RBE}\right)}{50.4\;\mathrm{Gy}\left(\mathrm{RBE}\right)}\approx 18\;\mathrm{Gy}\left(\mathrm{RBE}\right)$ isodose line. Typically, IL *V*_20 Gy(RBE)_ and *V*_18 Gy(RBE)_ only differ by about 1%–2% (see Fig. 2), again less than the RMSE of our model prediction. As a result, our model can be directly applied to all the common prescription dose levels, including sequential boost, while acknowledging the RMSE of 5%–6%.

A major portion of the cohort (80%) had nodal involvement, corresponding to a variety of target shapes on which our model was validated. None of the cases was partial breast irradiation, for which lung dose is usually not a clinical concern.

## 5 CONCLUSION

To conclude, we have established a contour‐based model that enable prediction of IL *V*_20 Gy(RBE)_ and *V*_5 Gy(RBE)_ for breast cancer patients treated with proton therapy. The model is clinically easy to implement and provides an estimate of the expected values prior to planning.

## CONFLICT OF INTEREST

No conflicts of interest.
